# The Effects of Partially Replacing Soybean Meal with Yeast Powder on the Growth Performance, Apparent Nutrient Digestibility, Serum Parameters, and Gut Microbiota of Weaned Pigs

**DOI:** 10.3390/ani16142176

**Published:** 2026-07-13

**Authors:** Jinkai Tang, Huatao Liu, Zhijin Hu, Zhangcheng Li, Zhihong Sun, Yetong Xu, Weizhong Sun, Jiaman Pang, Zhiru Tang

**Affiliations:** Animal Nutrition and Bio-Feed, Key Laboratory of Chongqing Education Commission of China, College of Animal Science and Technology, Southwest University, Chongqing 400715, China; 19330900225@163.com (J.T.); 18370732725@163.com (H.L.); hzj039@163.com (Z.H.); lzhangcheng@163.com (Z.L.); sunzh2002cn@aliyun.com (Z.S.); xuyetong@swu.edu.cn (Y.X.); swu2012@swu.edu.cn (W.S.); pangjm@swu.edu.cn (J.P.)

**Keywords:** yeast powder, weaned pigs, antioxidant capacity, intestinal flora

## Abstract

The aim of this experiment was to investigate the effects of partially replacing soybean meal with yeast powder in the diet on the growth performance, apparent nutrient digestibility, serum parameters, and gut microbiota of weaned pigs. A total of 64 commercial pigs of the ‘Duroc × Landrace × Large White’ crossbreed, with an average age of 50 days and a body weight of 19.8 ± 1.6 kg, were selected and randomly divided into four groups, each comprising 16 replicates. The pigs were fed either a control diet or experimental diets in which soybean meal was partially replaced by yeast powder at levels of 3% (LYP), 6% (MYP), and 9% (HYP), respectively, over a 26-day trial period. The results showed that replacing soybean meal with yeast powder significantly reduced the final body weight, average daily weight gain, average daily feed intake, and apparent digestibility of dry matter and crude ash in weaned pigs, while increasing the feed-to-gain ratio (*p* < 0.05); it also increased serum total antioxidant capacity and glutathione peroxidase activity (*p* < 0.05) and increased serum urea nitrogen and triglyceride levels, while reducing glucose and albumin levels in the HYP group (*p* < 0.05). Concurrently, the replacement of soybean meal with yeast powder reshaped the gut microbiota structure; the MYP group exhibited the highest α-diversity, with increased abundance of the Firmicutes phylum. The LYP group was specifically enriched with the *Streptococcus* and *Lactobacillus* genera, while the MYP group was specifically enriched with an unclassified genus within the family *Selenomonadaceae*, and the HYP group was enriched with the Actinobacteriota and *Clostridia_UCG_014*. Low- and medium-dose yeast powder supplementation was associated with enhanced antioxidant indices and distinct alterations in gut microbial composition compared with the control group; however, these changes occurred alongside reductions in growth performance; the optimal dose of yeast powder to replace soybean meal under the conditions of this trial requires further investigation.

## Simple Summary

Soybean meal is one of the most widely used protein sources in pig diets worldwide, but its increasing cost has encouraged the search for alternative protein ingredients. This study tested whether yeast powder—a safe and nutritious feed ingredient—could partly replace soybean meal for weaned pigs. We used 64 crossbred weaned pigs and divided them into four groups: a control group with regular feed and three test groups where 3%, 6%, and 9% of soybean meal was replaced with yeast powder. The trial lasted 26 days. We found that replacing soybean meal with yeast powder lowered pig growth and nutrient digestion across all replacement levels. However, the substitution improved the pigs’ antioxidant ability. Furthermore, medium and low doses of yeast powder supported healthier gut bacteria, whereas the high dose showed more pronounced negative effects. Overall, although replacing soybean meal with yeast powder reduced growth performance, low- and medium-level replacement improved antioxidant status and fecal microbiota composition. These findings provide a basis for further evaluation of yeast powder as an alternative protein ingredient in pig diets.

## 1. Introduction

With the increasing demand for high-quality protein feed ingredients in intensive pig production systems, soybean meal has long been the predominant protein source in swine diets because of its balanced amino acid profile, high digestibility, and excellent palatability. However, China’s heavy dependence on imported soybeans, coupled with fluctuations in global supply chains and rising market prices, has increased feed costs [1,2]. In addition, antinutritional factors present in soybean meal, including trypsin inhibitors, phytic acid, and oligosaccharides, may impair intestinal digestion and nutrient utilization, thereby affecting production performance [3]. Therefore, identifying sustainable and effective alternative protein sources to partially replace soybean meal has become an important research focus in pig nutrition.

Yeast powder, a high-quality microbial source of single-cell protein, is primarily produced from brewer’s yeast through processes such as liquid fermentation, separation, and drying. It typically contains 40–50% crude protein, has a balanced amino acid profile, is rich in lysine, threonine, and other key limiting amino acids for pigs, and contains virtually no antinutritional factors found in soybean meal. More importantly, yeast powder is rich in various bioactive substances, including β-glucans, mannan oligosaccharides, B vitamins, and functional peptides [4]. Among these, yeast β-glucan features a main chain composed of β-(1→3)-D-glucosidic bonds and side chains of β-(1→6)-D-glucosidic bonds. It is recognized by intestinal immune cells [5] and possesses biological functions such as regulating the intestinal microecological balance, enhancing the body’s immune and antioxidant capacities, and improving intestinal barrier function [6]. Therefore, yeast powder has potential as a functional feed ingredient in pig nutrition [7,8].

To date, there has been some research on the application of yeast-based ingredients in swine production. For example, Barducci et al. (2024) partially or completely replaced plasma protein powder with autolyzed yeast in weaned piglet diets and found that this significantly improved feed conversion efficiency, reduced the frequency of antibiotic use, and modulated the gut microbiota composition, increasing the relative abundance of beneficial bacteria such as *Lactobacillus* [8]. Boontiam et al. (2022) reported that adding 5–10% hydrolyzed yeast to the diet of early-weaned piglets linearly increased daily weight gain and feed intake and improved the apparent digestibility of crude protein and the ratio of villus height to crypt depth in the jejunum, while simultaneously enhancing IgA levels. It also reduced concentrations of pro-inflammatory cytokines (IL-1β, IL-6, and TNF-α) while promoting Lactobacillus proliferation and inhibiting *E. coli* counts [9]. However, these studies have primarily used yeast-derived products as test materials. Systematic testing and comprehensive evaluation of key indicators—such as production performance, serum biochemical parameters, gut health, nutrient digestibility, and immune function—in weaned pigs when whole-yeast powder directly replaces soybean meal at different ratios remain insufficient. The appropriate replacement levels and mechanisms of action require further clarification.

Based on this, the experiment substituted different amounts of yeast powder for soybean meal in the diet to examine its effects on the growth performance, nutrient digestibility, serum parameters, and gut microbiota of weaned pigs. The goal of the study is to establish a foundation for finding substitutes for soybean meal in diets and encouraging the use of yeast powder.

## 2. Materials and Methods

### 2.1. Materials

The yeast powder was purchased from Binzhou Furui Kang Feed Co., Ltd. (Binzhou, China). Commercial solvent-extracted soybean meal (44% crude protein) was used as the soybean meal source in the experimental diets, which were manufactured by a commercial feed mill. A total of 64 Duroc × Landrace × Large White weaned pigs were purchased from Chongqing Nongdianshan Agricultural Technology Development Co., Ltd. (Chongqing, China). In addition, all assay kits used in the experiment were purchased from Nanjing Jiancheng Bioengineering Institute (Nanjing, China).

### 2.2. Animals, Experiment Designs, Diets, Animal Feeding Management

This experiment employed a completely randomized design with a single factor. The trial lasted 26 days. A total of 64 healthy Duroc × Landrace × Large White three-way crossbred pigs with an initial body weight of 19.8 ± 1.6 kg (mean ± standard deviation), measured upon arrival at the experimental facility, were randomly divided into 4 groups, with 16 replicates per group and 1 pig per replicate. Four diets were used in the experiment: the CON group was fed a corn-soybean meal-based basal diet; the LYP group was fed a diet in which 3% of the soybean meal was replaced with yeast powder; the MYP group was fed a diet in which 6% of the soybean meal was replaced with yeast powder; and the HYP group was fed a diet in which 9% of the soybean meal was replaced with yeast powder. All diets were formulated according to NRC nutrient requirements for 20–40 kg weaned pigs [10]. A single-phase feeding strategy was adopted to maintain dietary consistency across treatments and to isolate the effects of yeast powder supplementation. The composition and nutritional levels of the diets for each experimental group are shown in Table 1. All experimental diets were provided in powder form.

The experiment was conducted at the Southwest University Experimental Pig Farm (Chongqing, China). The test animals were housed individually in pens. Prior to the experiment, the pens and equipment were cleaned and then disinfected with a 1% sodium hydroxide solution. After 24 h, they were rinsed with clean water, followed by a 48 h fumigation disinfection of the pens, feed troughs, feed buckets, and drinking water equipment using formalin and potassium permanganate (30 mL of formalin and 15 g of potassium permanganate per cubic meter). After ventilating the pens for five days, the experiment was initiated.

Pigs were fed three times daily at 7:30, 12:30, and 18:30 throughout the experimental period. Water was provided ad libitum. Daily management followed the Specification of Feeding and Management for Intensive Pig Farms [11]. Environmental conditions such as ventilation and air quality were monitored to maintain a clean and healthy housing environment. Pens were cleaned regularly and disinfected daily using a commercial disinfectant. Feed intake and residual feed were recorded daily for each replicate.

### 2.3. Sample Collection

Daily feed intake was recorded throughout the experiment. Weaned pigs were weighed in the early morning on an empty stomach on days 1 and 27 of the experiment. Average daily gain (ADG), average daily feed intake (ADFI), and feed conversion ratio (FCR) were calculated, and body length, height, and chest circumference were measured.

Starting on day 15 of the trial, four weaned pigs were randomly selected from each group, and 0.1% TiO_2_ was added to their feed as a chyme marker. On days 21–23, feces were collected once daily and mixed with 10% sulfuric acid (10 mL per 100 g of feces) to prevent evaporation and nitrogen loss. At the end of each collection period, fecal samples obtained from each replicate in each group were pooled and divided into subsamples, which were then frozen at −20 °C. The fecal subsamples were dried, ground, and sieved through a 1 mm sieve for nutritional analysis.

At the end of the experiment, five weaned pigs were randomly selected from each group, and 5 mL of blood was collected from the anterior vena cava into 10 mL heparinized vacuum blood collection tubes. The blood was centrifuged at 3000 rpm for 10 min at 4 °C, and the serum was collected, aliquoted into 1.5 mL centrifuge tubes, and stored at −80 °C. Rectal fecal samples were collected using rectal swabs, placed in sterile, enzyme-free centrifuge tubes, and analyzed for microbial flora. The samples were frozen in liquid nitrogen and stored at −80 °C.

### 2.4. Composition of the Basic Diet

Collect feed samples; dry them at 105 °C to constant weight; grind them through a 1.0 mm sieve; and store them at 4 °C until analysis. The contents of crude protein (CP), calcium (Ca), total phosphorus (TP), crude fiber (CF), neutral detergent fiber (NDF), acid detergent fiber (ADF), and amino acids (AAs) were determined according to the standard methods of the Association of Official Analytical Chemists [12]. Available phosphorus (AP) content was determined using spectrophotometry. Digestible energy (DE) values were calculated based on the measured composition of each ingredient, in conjunction with the digestible energy coefficients for weaned pigs published by the NRC [10].

### 2.5. Growth Performance

The growth performance of the pigs is calculated using the following formula based on their initial weight, final weight, and feeding intake during the experimental period:

Weight gain (kg) = final weight (kg) − initial weight (kg);(1)

Average daily gain (ADG) = weight gain (g)/day (d);(2)

Average daily feed intake (ADFI) = feed intake during the trial period (g)/day (d);(3)

Feed conversion ratio (FCR) = feed consumption/weight gain.(4)

### 2.6. Apparent Nutrient Digestibility

Samples of feed and feces were collected separately, dried in an oven at 65 °C, and then ground to pass through a 40-mesh sieve. Subsequently, CP and ash contents were determined separately in accordance with national standards [13,14]. The apparent nutrient digestibility was calculated using the following formula:

Digestibility (%) = 100% − (titanium dioxide content in feed × nutrient content in feces)/(titanium dioxide content in feces × nutrient content in feed) × 100%.

### 2.7. Serum Index

Using the appropriate commercial assay kit (Nanjing Jiancheng Biotechnology Research Institute, Nanjing, China), serum calcium (Ca), serum phosphorus (P), serum calcitonin (CT), parathyroid hormone (PTH), alkaline phosphatase (ALP), hydroxyproline (HYP), blood urea nitrogen (BUN), albumin (ALB), triglycerides (TG), glucose (GLU), total protein (TP), total cholesterol (TC), total serum superoxide dismutase (T-SOD), total antioxidant capacity (T-AOC), catalase (CAT), glutathione peroxidase (GSH-Px), and malondialdehyde (MDA) levels were measured in serum.

### 2.8. Gut Microbiome Analysis

Rectal 16S rDNA microbial analysis was performed by Hangzhou Lianchuan Biotechnology Co., Ltd. (Hangzhou, China). The company extracted sample DNA, performed 16S rDNA PCR amplification, constructed an amplicon library for paired-end sequencing, and completed data collection and bioinformatics analysis of the 16S rDNA V3–V4 regions from the rectum using the Illumina sequencing platform (San Diego, CA, USA). After on-platform sequencing was completed, the raw off-platform data (RawData) was assembled into paired-end data using the overlapping method, followed by quality control and chimera filtering to obtain high-quality CleanData. Subsequently, an OTU-like (Operational Taxonomic Units) table was constructed using the concept of ASVs (Amplicon Sequence Variants), yielding the final ASV feature table and feature sequences. This was followed by further diversity analysis, species classification annotation, and differential analysis using fqtrim version 0.94, Vsearch version 2.3.4, and R software version 3.5.2.

### 2.9. Statistical Analysis

Data were analyzed using one-way analysis of variance (ANOVA) and covariance analysis in SPSS 29.0 (SPSS, Inc., Chicago, IL, USA), and Duncan’s multiple-range test was used to compare significance. Orthogonal polynomial analysis was used to examine linear and quadratic relationships. Significance was set at *p* < 0.05. All data are presented as means ± standard error of the mean (SEM).

## 3. Results

### 3.1. Growth Performance

As shown in Table 2, there were no significant differences in the initial body weights of the weaned pigs across the groups (*p* > 0.05). Compared with the CON group, the FBW and ADG of the LYP, MYP, and HYP groups were significantly lower (*p* < 0.01), and these values decreased as the level of yeast powder replacing soybean meal increased. Regarding ADFI, there were no significant differences among the CON, LYP, and MYP groups (*p* > 0.05), whereas the ADFI in the HYP group was significantly lower (*p* < 0.01). Compared with the CON group, the FCR in the experimental groups increased significantly as the level of yeast powder replacing soybean meal increased (*p* < 0.01). Final body weight, average daily weight gain, average daily feed intake, and feed-to-meat ratio exhibited linear or quadratic relationships with the amount of yeast powder substituted in the diet (linear, *p* < 0.05; quadratic, *p* < 0.05).

### 3.2. Body Type

As shown in Table 3, there was no significant difference in body height between the CON group and the LYP group (*p* > 0.05), while the MYP and HYP groups exhibited significantly lower body heights than the CON group (*p* < 0.05), with body height decreasing as the replacement level increased. Regarding body length, the LYP and MYP groups were significantly longer than the other two groups (*p* < 0.01), with no significant difference between the two; the HYP group was significantly shorter than both the LYP and MYP groups (*p* < 0.01); the CON group had the shortest body length, significantly shorter than the other groups (*p* < 0.01). Regarding chest circumference, the CON group was significantly larger than the other groups (*p* < 0.01), while the LYP group had a significantly greater chest circumference than the MYP and HYP groups (*p* < 0.01); there was no significant difference in chest circumference between the MYP and HYP groups (*p* > 0.05). Body height, body length, and chest circumference exhibited a linear or quadratic relationship with the yeast powder replacement level in the diet (linear, *p* < 0.05; quadratic, *p* < 0.05).

### 3.3. Apparent Nutrient Digestibility

As shown in Table 4, the CON group had the highest CP digestibility, which was significantly higher than that of the MYP group (*p* < 0.05); there were no significant differences between the other groups. The DM digestibility of the CON group was extremely significantly higher than that of all treatment groups (*p* < 0.01). There were no significant differences between the LYP, MYP, and HYP groups, and digestibility decreased as the level of yeast powder replacing soybean meal increased. The ash digestibility of the CON group was significantly higher than that of all experimental groups (*p* < 0.05), and the ash digestibility of the HYP group was significantly higher than that of the MYP group (*p* < 0.05). There was a linear or quadratic relationship between DM digestibility and the amount of yeast powder substituted in the diet (linear, *p* < 0.05; quadratic, *p* < 0.05), while there was a quadratic relationship between ash digestibility and the amount of yeast powder substituted in the diet (quadratic, *p* < 0.05).

### 3.4. Serum Calcium and Phosphorus Metabolism

As shown in Table 5, there was no significant difference in serum Ca and P concentrations among the experimental groups (*p* > 0.05). Serum hydroxyproline levels were significantly higher in the CON and LYP groups than in the HYP group (*p* < 0.05), while there was no significant difference between the MYP and HYP groups. The CT content showed an increasing trend with increasing levels of yeast powder replacement, and the HYP group had significantly higher CT levels than the CON and LYP groups (*p* < 0.05). PTH levels showed an opposite trend; the CON group had the highest PTH content, which decreased sequentially in the LYP, MYP, and HYP groups. The CON and LYP groups exhibited significantly higher PTH levels than the HYP group (*p* < 0.05). ALP activity increased with increasing levels of yeast powder substitution; ALP activity in the HYP group was significantly higher than that in the CON and LYP groups (*p* < 0.05). Notably, HYP content, CT levels, PTH content, and ALP activity all exhibited a significant linear response to the amount of yeast powder substituted in the diet (*p* < 0.01). The patterns observed in Table 5 suggest a coordinated regulation of bone metabolism-related markers in response to dietary yeast powder supplementation. The inverse relationship between PTH and CT levels, together with the dose-dependent increase in ALP activity, indicates that yeast powder may influence bone metabolic activity despite no significant alterations in circulating mineral concentrations. These changes may reflect an enhanced bone turnover status under increasing levels of soybean meal replacement. The observed responses suggest that yeast powder supplementation modulates bone metabolism and mineral utilization efficiency; however, further studies are required to clarify the underlying mechanisms.

### 3.5. Serum Biochemical Indexes

As shown in Table 6, there were no significant differences in serum TP and T-CHO concentrations among the experimental groups (*p* > 0.05). Serum BUN levels were significantly higher in the LYP, MYP, and HYP groups compared to the CON group (*p* < 0.05), with the MYP group having the greatest BUN concentration. Serum GLU levels in the CON group were significantly higher than those in the other three groups (*p* < 0.05). Serum ALB levels in the CON and LYP groups were significantly elevated compared to the HYP group (*p* < 0.05). TG levels increased as the level of yeast powder replacing soybean meal increased; specifically, TG levels in the HYP group were significantly higher than those in the CON and LYP groups, and TG levels of the MYP group were significantly higher than those of the CON group (*p* < 0.05). Notably, BUN, GLU, ALB, and TG levels all exhibited a significant linear response to the amount of yeast powder substituted in the diet (*p* < 0.01). The trends observed in Table 6 provide preliminary insights into metabolic responses to dietary yeast powder supplementation. The linear increasing trend in BUN levels suggests a potential alteration in protein metabolism, indicating that a higher replacement of soybean meal with yeast powder might lead to increased amino acid catabolism and a relative reduction in nitrogen utilization efficiency. Furthermore, the linear increase in TG combined with the decrease in GLU may indicate a shift in systemic energy metabolism, potentially reflecting an increased reliance on lipid metabolism relative to glucose utilization under higher levels of yeast powder substitution. However, these interpretations should be considered as exploratory and warrant further mechanistic investigation.

### 3.6. Antioxidant Capacity

As shown in Table 7, T-AOC activity increased with increasing levels of yeast powder substitution; the HYP and MYP groups exhibited significantly higher T-AOC activity than the CON and LYP groups (*p* < 0.05). GSH-Px activity in the HYP group was significantly higher than that in the CON, LYP, and MYP groups (*p* < 0.05), with no significant differences observed among the latter three groups. No significant differences in T-SOD activity, CAT activity, or MDA content among the experimental groups (*p* > 0.05). Regarding the dose–response relationship, T-AOC activity exhibited a significant linear response to the dietary inclusion level of yeast powder (*p* < 0.01), while GSH-Px activity showed a significant quadratic response (*p* < 0.01). The response patterns observed in Table 7 suggest that dietary yeast powder supplementation differentially influences non-enzymatic and enzymatic antioxidant systems. The linear increase in T-AOC indicates a dose-related enhancement in total antioxidant capacity. Meanwhile, the linear increase in GSH-Px suggests a specific regulatory pattern, where the activity of GSH-Px is enhanced in a dose-dependent manner. These findings suggest that the antioxidant benefits of yeast powder are dose-dependent and involve distinct regulatory pathways within the antioxidant defense system.

### 3.7. Analysis of Gut Microbiota Composition and Diversity

**Figure 1:**
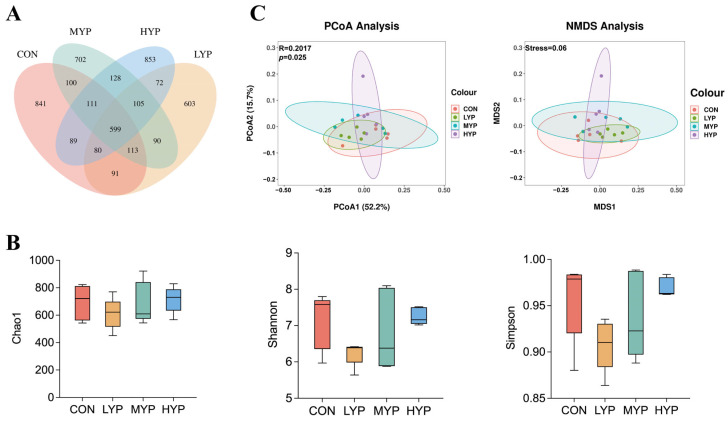
Effects of yeast powder on gut microbial composition and diversity in weaned pigs: (**A**) Venn diagram based on amplicon sequence variants (ASVs), where different colors represent different experimental groups and numbers indicate the numbers of shared and unique ASVs among groups; (**B**) alpha diversity shown by Chao1, Shannon, and Simpson indices; (**C**) principal coordinate analysis (PCoA) and non-metric multidimensional scaling (NMDS) based on weighted UniFrac distance. CON, basal diet group; LYP, 3% yeast powder group; MYP, 6% yeast powder group; HYP, 9% yeast powder group. Each group contains 5 replicates (*n* = 5).

**Figure 2:**
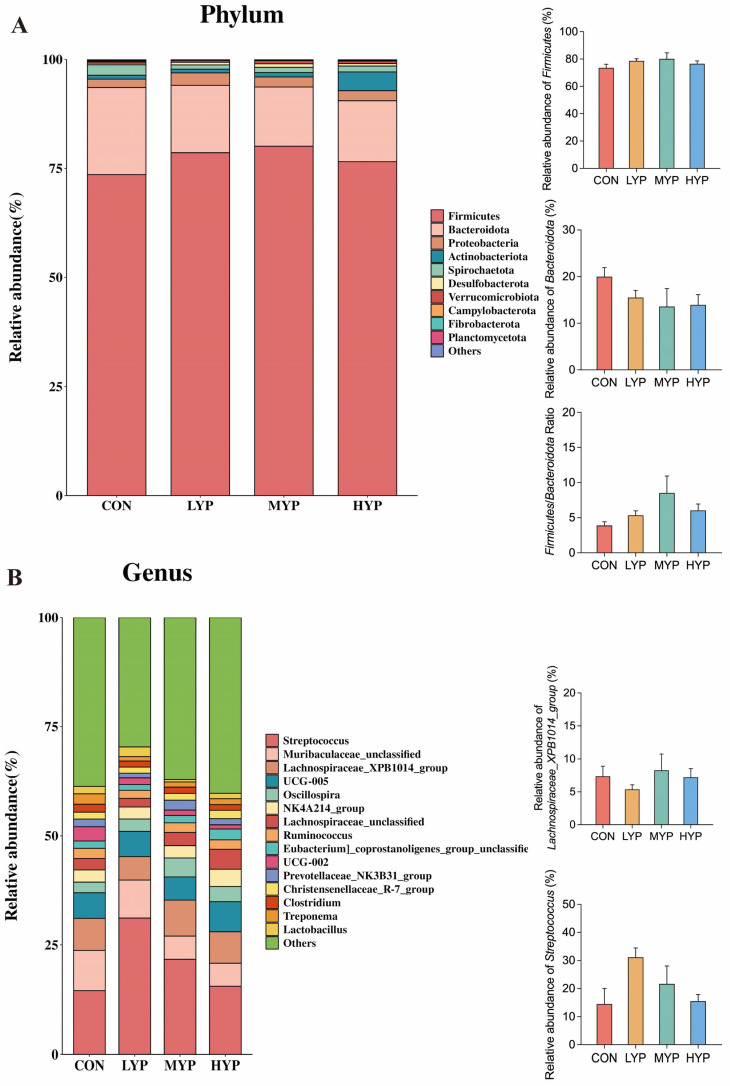
(**A**) Relative abundance of gut microbiota at the phylum level; (**B**) relative abundance of gut microbiota at the genus level. CON, basal diet group; LYP, 3% yeast powder group; MYP, 6% yeast powder group; HYP, 9% yeast powder group. Each group contains 5 replicates (*n* = 5).

**Figure 3:**
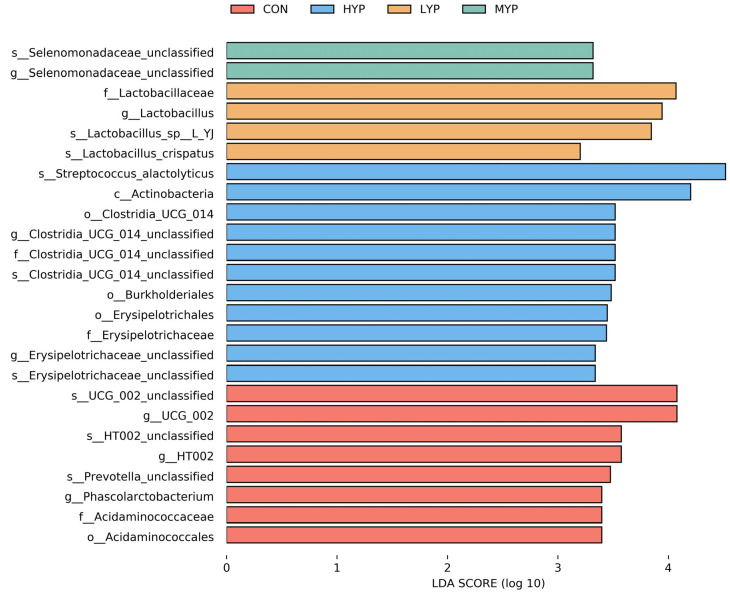
Differentially enriched taxa identified by linear discriminant analysis effect size (LEfSe) (LDA ≥ 3.0, *p* < 0.05). CON, basal diet group; LYP, 3% yeast powder group; MYP, 6% yeast powder group; HYP, 9% yeast powder group. Each group contains 5 replicates (*n* = 5).

As shown in Figure 1, the Venn diagram reveals that there are 599 shared ASVs across all groups. The CON, LYP, MYP, and HYP groups had 841, 603, 702, and 853 unique ASVs, respectively (Figure 1A). Analysis of gut microbial diversity and richness revealed that the MYP group had the highest microbial richness as measured by the Chao1 and Shannon indices, while the LYP group had the lowest; the CON and HYP groups exhibited intermediate levels of richness. Furthermore, the Simpson index was higher in the CON and HYP groups, lowest in the LYP group, and intermediate in the MYP group (Figure 1B). Principal Coordinate Analysis (PCoA) and Non-Metric Multidimensional Scaling (NMDS) revealed a statistically significant separation in microbial community composition among the different experimental groups (R = 0.2017, *p* = 0.025; Figure 1C). As shown in the PCoA plot (Stress = 0.06), the samples from the treatment groups exhibited distinct clustering patterns relative to the CON group. These results indicate that the dietary yeast powder supplementation effectively induced shifts in the overall structure and composition of the gut microbiota. Firmicutes and Bacteroidota were the dominant phyla in all four groups (Figure 2A), accounting for more than 90% of total microbial abundance and constituting the core steady-state structure of the gut microbiota; Proteobacteria, Actinobacteriota, Spirochaetota, and others were low-abundance rare phyla. Compared with the CON group, the relative abundance of Firmicutes was elevated in all experimental groups, whilst that of Bacteroidota was reduced. This shift naturally resulted in an increased Firmicutes-to-Bacteroidota (F/B) ratio in the yeast powder-supplemented groups, a microbial signature frequently associated with an enhanced capacity for energy harvest and improved nutrient absorption in the host. As shown in Figure 2B, at the genus level, among the annotated genera, *Streptococcus*, Muribaculaceae_unclassified, and Lachnospiraceae_XPB1014_group were the core dominant genera, constituting the primary functional framework of the gut microbiota. The relative abundance of *Streptococcus* was higher in all experimental groups than in the CON group, exhibiting a pattern of enrichment at low doses followed by a decline at medium and high doses. The dose-dependent modulation of these core genera suggests that yeast powder acts selectively on the microbial ecosystem, potentially optimizing the balance between complex carbohydrate fermenters and lactic acid-producing bacteria. Linear Discriminant Analysis Effect Size (LEfSe) identified multiple differentially enriched taxa (LDA ≥ 3.0; *p* < 0.05). Specifically, the CON group was predominantly enriched with characteristic genera such as UCG-002, HT002, and *Phascolarctobacterium*; the LYP group was specifically enriched with *Lactobacillus*; the MYP group was significantly enriched with Selenomonadaceae_unclassified; and the HYP group was significantly enriched with Actinobacteria at the phylum level while also showing enrichment of genera related to Clostridia_UCG_014 (Figure 3). This biomarker transition reveals a highly specific microecological evolution driven by the dietary intervention. The distinct enrichment of Lactobacillus in the LYP group indicates a strong promotion of beneficial bacteria known to lower gut pH and inhibit opportunistic pathogens. Furthermore, the progressive enrichment of Selenomonadaceae and Clostridia lineages in the higher-dose groups highlights an augmented functional potential for dietary fiber degradation and short-chain fatty acid (SCFA) production. Collectively, these taxonomic shifts indicate that dietary yeast powder altered the composition of the gut microbial community.

## 4. Discussion

**Figure 4:**
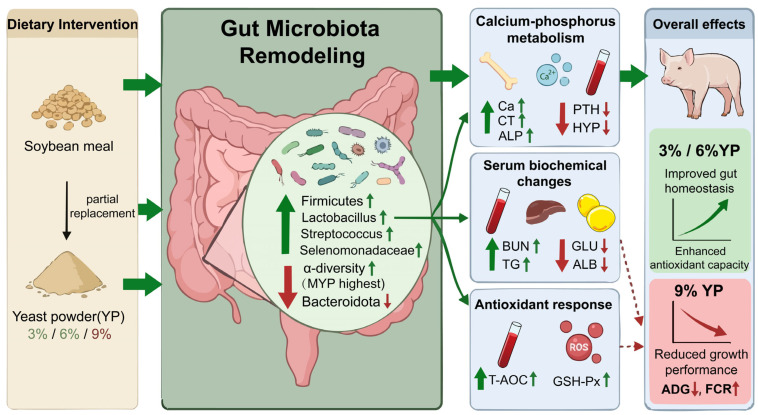
Mechanism diagram of the effects of partial replacement of soybean meal with yeast powder on weaned pigs. Green arrows indicate positive regulatory effects, whereas red arrows indicate negative effects. Upward and downward arrows represent increases and decreases in the corresponding parameters, respectively. Different colors represent different functional modules.

To summarize the multifaceted effects of yeast powder supplementation, we constructed a mechanistic diagram (Figure 4) illustrating the dose-dependent regulatory pathways centered on gut microbiota remodeling.

The results of this trial indicate that replacing a large proportion of soybean meal with yeast powder in the diet significantly inhibits the growth performance and body conformation of weaned pigs, and this effect is clearly dose-dependent. In terms of growth performance, compared with the control group, both FBW and ADG were significantly reduced in the low-, medium-, and high-dose yeast powder groups, while FCR increased significantly as the level of yeast powder replacing soybean meal increased. ADFI decreased significantly only in the high-dose group, while there were no significant differences between the low- and medium-dose groups and the control group. Regarding body conformation, there was no significant difference in body height between the control and LYP groups; the MYP and HYP groups exhibited significantly lower body heights than the CON group, with a decreasing trend as the replacement level increased. Regarding body length, the LYP and MYP groups were significantly longer than the CON and HYP groups, with the CON group having the shortest body length; regarding chest circumference, the CON group was significantly larger than all experimental groups, the LYP group was significantly larger than the MYP and HYP groups, and there was no significant difference between the latter two groups. The above results are consistent with previous reports [15]. A meta-analysis by Choi and Kim (2023) further clarified that the optimal inclusion level of yeast β-glucan in diets for weaned piglets is only 50 mg/kg (0.005%), as excessive supplementation inhibits growth due to overstimulation of the intestinal immune system and increased maintenance energy expenditure [5]. In the standard feed-grade yeast powder used in this trial, β-glucan consists of a main chain of β-(1→3)-D-glucosidic bonds and side chains of β-(1→6)-D-glucosidic bonds, constituting a structural polysaccharide that cannot be broken down by endogenous digestive enzymes in animals [16]. When 3% yeast powder was added to the diet, the actual β-glucan content reached 0.15–0.3%, far exceeding the optimal threshold, which was the primary cause of abnormal growth performance and body conformation. Previous studies have shown that the structural characteristics of yeast β-glucan are closely related to its physiological functions; this branched β-type linkage allows it to form a stable triple-helix conformation [17,18], which cannot be degraded by endogenous enzymes such as amylase and saccharolysin in the pig’s digestive tract. Similarly, relevant research indicates that excessive supplementation of yeast β-glucan leads to decreased nutrient digestibility and a higher feed-to-gain ratio in piglets [5].

This study found that the DM digestibility in the CON group was significantly higher than that in all treatment groups (*p* < 0.01) and that it decreased as the level of yeast powder replacing soybean meal increased; this is partially consistent with the findings of Øverland and Skrede (2017). They found that although yeast powder can enhance intestinal digestive enzyme activity, high-dose supplementation reduces DM digestibility, mainly owing to the physical barrier formed by yeast cell wall polysaccharides such as β-glucans and mannans [15]. The decrease in DM digestibility observed in this study may be attributed to cell wall components in the yeast powder (such as β-glucans and mannan oligosaccharides) increasing the dietary fiber content, thereby increasing fecal excretion and leading to a reduction in apparent DM digestibility. Dietary fiber can affect the bioavailability of minerals by forming insoluble complexes with mineral ions such as calcium, magnesium, and zinc or by altering the physicochemical properties of the chyme [19]. In this trial, the ash digestibility of the CON group was significantly higher than that of all treatment groups (*p* < 0.01), indicating that the basal soybean meal diet is more conducive to the overall digestion and absorption of minerals, whereas the replacement of soybean meal with yeast powder increased the fiber content of the diet (e.g., β-glucans and mannan oligosaccharides in yeast cell walls), which may have bound with certain mineral elements in the intestine to form insoluble complexes or accelerated intestinal motility to shorten the absorption time of mineral elements, thereby leading to an overall decrease in ash digestibility across all experimental groups.

Serum Ca and P levels are important indicators of Ca and P nutritional status and also reflect Ca and P metabolism in the bones; a slight increase in blood Ca and P levels within the normal range is beneficial for animal growth [20,21]. The results of this study indicate that replacing soybean meal with yeast powder increases serum calcium levels but does not significantly affect serum phosphorus levels. As key antagonistic hormones in calcium metabolism, the synergistic regulation of PTH and CT constitutes a classic pathway for maintaining calcium homeostasis in animals [22,23,24]. Li et al. (2022) clearly demonstrated in a blue fox study that serum CT and PTH levels exhibit an inverse relationship with dietary calcium concentration. Elevated CT maintains calcium homeostasis by inhibiting osteoclast activity and inducing calcium deposition in bone tissue, while reduced PTH decreases bone calcium mobilization; this is fully consistent with the ‘elevated CT + reduced PTH’ results observed in this study. The study also found that CT interacts with VD_3_ to jointly regulate intestinal calcium absorption and bone calcium deposition, suggesting that yeast extract in this study may indirectly enhance vitamin D_3_ utilization by improving the intestinal environment, thereby synergistically activating the CT-mediated bone calcium deposition regulatory pathway [22]. As a key marker of bone formation, ALP activity directly reflects osteoblast proliferation and the efficiency of bone mineralization [25]. In this experiment, serum ALP activity increased as the level of yeast powder replacing soybean meal rose, indicating that yeast powder, by improving the efficiency of intestinal calcium and phosphorus absorption, provides osteoblasts with sufficient nutritional substrates, activates the ALP-mediated bone mineralization pathway, and ultimately enhances bone formation.

Serum biochemical parameters reflect the body’s physiological and metabolic response to diet and, to a certain extent, provide an indication of the animal’s nutritional metabolism and health status [7,26]. In this study, BUN levels in all experimental groups were higher than those in the CON group, and BUN levels in the MYP and HYP groups were significantly higher than those in the CON group. This suggests that following the replacement of soybean meal with yeast powder, protein catabolism was enhanced or nitrogen utilization efficiency was reduced, which may indicate a decrease in the bioavailability of dietary protein, which is consistent with the observed trend of reduced CP digestibility in this study, further supporting the hypothesis that the crude fiber component of yeast cell walls influences the absorption of nutrients and minerals. GLU levels were lower in all experimental groups compared to the CON group, with the LYP and HYP groups showing significantly lower GLU levels than the CON group. This suggests that replacing soybean meal with yeast powder restricted glucose availability or altered systemic energy balance, which is consistent with the lower dry matter digestibility and reduced growth performance observed across the treatment groups [27]. As a key indicator reflecting hepatic synthetic function and the body’s nutritional status, serum ALB levels in the HYP group were extremely significantly lower than those in the CON and LYP groups (*p* < 0.01), while there were no significant differences in TP levels across the groups. This suggests that replacing soybean meal with high levels of yeast powder may have a mild effect on hepatic albumin synthesis but does not alter the body’s TP levels. This indicates that the substitution did not cause a severe imbalance in the body’s protein nutrition but rather had a certain negative impact on nitrogen metabolism efficiency only at medium-to-high doses. TG levels are an important indicator of fat absorption and lipid metabolism status [28,29]. In this study, TG levels increased as the level of yeast powder replacing soybean meal increased; TG levels in the HYP group were significantly higher than those in the CON and LYP groups, suggesting that replacing soybean meal with yeast powder may promote lipid synthesis or inhibit lipid catabolism.

Serum antioxidant markers are key biological indicators that reflect the body’s redox homeostasis, antioxidant defense capacity, and the extent of lipid peroxidation damage; indeed, the body’s antioxidant status has become an important indicator for assessing meat quality and is used to evaluate the risk of food deterioration caused by oxidative stress [7,30,31]. T-AOC is a comprehensive indicator of the body’s overall antioxidant defense level, and its activity directly reflects the body’s overall capacity to scavenge free radicals and resist oxidative stress. The results of this study show that, compared with the CON group, serum T-AOC activity was significantly higher in the medium- and high-level yeast powder-replaced soybean meal groups (MYP, HYP) (*p* < 0.01), with all experimental groups exhibiting a dose-dependent increase as the level of yeast powder replacing soybean meal increased. These results indicate that yeast powder can effectively enhance the overall antioxidant capacity of weaned pigs, with a more pronounced synergistic effect at higher doses; the core mechanism is closely related to the active components in yeast powder, such as β-glucan and mannan oligosaccharides. Li et al. (2016) confirmed in a broiler study that β-glucans in yeast cell walls (YCW) can significantly enhance the antioxidant capacity of the intestinal mucosa, reduce the accumulation of lipid peroxidation products, and thereby improve the oxidative stability of meat [32]. Hou et al. (2020) further found that selenium-enriched Saccharomyces cerevisiae, by activating the glutathione and thioredoxin systems, significantly reduced water loss and MDA content in broiler chicken muscle while enhancing antioxidant enzyme activity [33], confirming a direct link between improved antioxidant status and enhanced meat quality, which provides a possible explanation for the increased T-AOC observed in the present study. In this study, only the HYP group exhibited significantly higher serum GSH-Px activity than the CON, LYP, and MYP groups (*p* < 0.01), with no significant differences observed among the remaining groups. This suggests that the regulation of GSH-Px activity by yeast powder exhibits a distinct dose-threshold effect and that only at high replacement levels can the active components in yeast powder fully activate the synthesis and secretion of GSH-Px. T-SOD and CAT are core components of the antioxidant enzyme system, responsible for protecting the body from oxidative damage caused by ROS [34], while MDA is the end product of lipid peroxidation; its concentration not only reflects the extent of oxidative damage in the body but is also a key indicator for assessing oxidative deterioration in meat products. The results of this study show that there were no significant differences in T-SOD and CAT activity or MDA content among the experimental groups (*p* > 0.05), suggesting that yeast powder exerts a certain degree of selectivity in modulating the antioxidant system of weaned pigs. Yeast and its active components may be more inclined to exert their antioxidant effects via the selenium-dependent GSH-Px pathway rather than the SOD/CAT pathway; a similar phenomenon was also observed in the study by Czech et al. (2020) [35]. Furthermore, as there were no significant differences in MDA levels between the groups, combined with the significantly elevated T-AOC and enhanced GSH-Px activity, this indicates that replacing soybean meal with yeast powder did not cause significant lipid peroxidation damage. The body’s antioxidant capacity was sufficient to scavenge excess reactive oxygen species generated by metabolism and maintain redox balance.

The gut microbiota is crucial to animal health, supporting nutrient absorption, immune function, and overall well-being [7]. The gut microbiota maintains host health by aiding the body in digesting and breaking down complex food molecules, thereby enhancing overall gut function [36]. The composition and diversity of the gut microbiota are largely influenced by dietary intake, making diet one of the most critical environmental factors shaping the microbial community [37]. Gut microbial diversity is typically measured using α-diversity indices, with the Chao1 index reflecting community richness and the Shannon and Simpson indices reflecting community diversity [38]. In this study, there were no significant differences in either α-diversity, indicating that dietary yeast powder did not markedly disrupt overall microbial diversity [39]; this phenomenon was similarly observed in the study by De La Guardia Hidrogo et al. (2025) [40]. Analysis of the gut microbiome revealed that, at the phylum level, Firmicutes and Bacteroidota were the predominant phyla observed across all groups, consistent with the findings of Barducci et al. [6]. Firmicutes is a key phylum in the gut responsible for producing short-chain fatty acids (SCFAs), which aid in the degradation of fiber and starch [41] and promote food digestion and microecological balance [42]. In this study, the MYP group exhibited the highest Firmicutes abundance, followed by the LYP and HYP groups, with the CON group showing the lowest abundance. The abundance exhibited a trend of initially increasing and then decreasing as the level of yeast powder replacing soybean meal increased, reaching a peak at the medium dose. The increase in Firmicutes abundance indicates that the medium dose of yeast powder can optimize the substrate environment, promote the proliferation of metabolically active bacteria within Firmicutes, and enhance the gut’s capacity to produce SCFAs through fermentation. Bacteroidota are primarily involved in the degradation and metabolism of complex polysaccharides and non-starch polysaccharides in the diet and are a key phylum for the utilization of intestinal polysaccharides. The results of this study show that the Bacteroidota abundance was highest in the CON group, with all experimental groups having lower Bacteroidota abundance than the CON group, and the MYP group having the lowest Bacteroidota abundance. This change may be related to alterations in the carbohydrate composition of the diet following the replacement of soybean meal with yeast powder: components in yeast powder, such as β-glucans and mannan oligosaccharides, act as specific substrates that preferentially support the proliferation of functional Firmicutes, leading to a reduction in available substrates for Bacteroidota and consequently inhibiting their abundance. A study by Barducci et al. (2024) found that feeding piglets a diet containing yeast autolysate enriches functional Firmicutes, which indirectly affects Bacteroidota abundance by competing for nutritional space and substrates [6]. At the genus level, the LYP group exhibited the highest abundance of *Streptococcus* in the gut, with all experimental groups showing higher *Streptococcus* abundance than the CON group. *Streptococcus* is an important commensal genus in the porcine gut, playing a significant role in nutrient digestion, vitamin synthesis, and the maintenance of gut microecology [43]. Components in yeast powder, such as β-glucan, provide suitable growth substrates for *Streptococcus*, promoting its colonization and proliferation. The highest enrichment of *Streptococcus* was observed in the LYP group, indicating that replacing soybean meal with low-dose yeast powder promotes *Streptococcus* proliferation and optimizes the functional structure of the gut microbiota. As the yeast powder dose increased, the abundance of *Streptococcus* gradually declined, suggesting that replacing soybean meal with excessively high doses of yeast powder alters the intestinal substrate environment, thereby reducing the proliferative promotion effect on this genus. LEfSe analysis revealed that the CON group was characterized by UCG-002, HT002, and *Phascolarctobacterium* as core differential bacterial communities, all of which are characteristic commensal bacteria under the basal diet, primarily maintaining basic intestinal fermentation functions. In the LYP group, the differential marker was *Lactobacillus*, with probiotic enrichment as the primary characteristic; the MYP group specifically enriched *Selenomonadaceae_unclassified*, a group of key lactic acid-utilizing and short-chain fatty acid-producing bacteria capable of optimizing intestinal energy metabolism. In the HYP group, the differential microbial communities were Actinobacteriota and *Clostridia_UCG_014*, which are involved in immune regulation and intestinal barrier repair, respectively. A comprehensive analysis of the differential microbial community characteristics reveals that the MYP and LYP groups demonstrated superior overall effects on gut microbial regulation compared to the CON and HYP groups.

Finally, it is important to acknowledge that the relatively small sample size used for serum biochemical and gut microbiota analyses (*n* = 5 per group) represents a limitation of the present study. Although significant differences were observed in several serum antioxidant indices and microbial biomarkers among the treatment groups, the limited sample size may have reduced the ability to detect more subtle biological effects and may not fully capture inter-individual variability within the population. Therefore, future studies involving larger cohorts are needed to validate the present findings and further elucidate the systemic and gut microbial responses to dietary yeast powder supplementation in weaned pigs.

## 5. Conclusions

This study demonstrated that partial replacement of soybean meal with yeast powder affected growth performance, nutrient digestibility, serum parameters, and gut microbial composition in weaned pigs. All tested replacement levels reduced growth performance and certain nutrient digestibility indices. However, dietary yeast powder increased serum antioxidant indices and was associated with shifts in gut microbial composition. These findings indicate that yeast powder may influence antioxidant status and gut microbial profiles beyond its nutritional contribution as a protein ingredient; however, under the conditions of the present study, yeast powder replacement did not improve growth performance. Further studies are required to optimize dietary formulations and clarify the mechanisms underlying these responses.

## Figures and Tables

**Table 1:** The ingredients and nutritional levels of diets.

Ingredients	Content (%)				Nutritional Level ^2^	Content			
	CON	LYP	MYP	HYP		CON	LYP	MYP	HYP
Corn	74.22	72.98	69.42	68.21	DE (MJ/kg)	14.30	14.25	14.25	14.28
Soybean meal, 44% CP	15.23	11.43	7.32	3.74	CP (%)	16.05	16.08	16.07	16.10
Yeast powder	0.00	3.00	6.00	9.00	Ca (%)	0.70	0.67	0.67	0.70
Fish meal	4.00	4.00	4.00	4.00	Total P (%)	0.58	0.58	0.59	0.63
Rice bran	1.00	3.44	7.98	9.43	Available P (%)	0.33	0.31	0.28	0.30
Soybean oil	2.27	2.09	2.32	2.55	crude fiber (%)	2.16	2.08	2.05	1.92
Calcium hydrophosphate	0.84	0.64	0.39	0.44	NDF (%)	9.20	9.31	9.64	9.56
Limestone	0.78	0.82	1.01	1.08	ADF (%)	3.60	3.58	3.75	3.63
Sodium chloride	0.30	0.30	0.30	0.30	Lysine (%)	0.99	0.98	0.98	0.97
Lysine hydrochloride	0.26	0.24	0.22	0.18	Methionine (%)	0.32	0.31	0.32	0.32
DL-Methionine	0.03	0.01	0.01	0.01	Threonine (%)	0.65	0.62	0.62	0.64
Threonine	0.06	0.02	0.01	0.01	Tryptophan (%)	0.19	0.19	0.17	0.17
L-Trp	0.02	0.03	0.03	0.04					
Premix ^1^	1.00	1.00	1.00	1.00					
Total	100.00	100.00	100.00	100.00					

^1^ Premix provided the following per kilogram of diet: vitamin A, 2050 IU; vitamin D_3_, 220 IU; vitamin E, 20 IU; vitamin K_3_, 0.6 mg; vitamin B_6_, 2.0 mg; vitamin B_12_, 20 μg; riboflavin, 4.0 mg; thiamine, 1.8 mg; niacin, 20 mg; pantothenic acid, 13 mg; folacin, 0.45 mg; biotin, 0.09 mg; choline chloride, 800 mg; wheat bran, 2.29 g; antioxidant, 200 mg; mold inhibitor, 50 mg; medical stone, 5.94 g; Cu, 6 mg; Fe, 90 mg; Zn, 90 mg; Mn, 4 mg; I, 0.14 mg; Se, 0.3 mg. ^2^ DE is the calculated value, and the others are measured values. Abbreviations: DE, digestible energy; CP, crude protein.

**Table 2:** Effects of replacing soybean meal with yeast powder on the growth performance of weaned pigs.

Items	Treatments				SEM	*p*-Value		
	CON	LYP	MYP	HYP		ANOVA	Linear	Quadratic
IBW/kg	19.59	19.96	19.89	19.94	0.196	0.908	0.581	0.797
FBW/kg	38.46 ^a^	36.23 ^b^	34.06 ^c^	31.37 ^d^	0.413	<0.01	<0.01	<0.01
ADG/kg	0.73 ^a^	0.63 ^b^	0.55 ^c^	0.44 ^d^	0.016	<0.01	<0.01	<0.01
ADFI/kg	1.65 ^a^	1.65 ^a^	1.62 ^a^	1.58 ^b^	0.007	<0.01	<0.01	<0.01
FCR	2.30 ^d^	2.68 ^c^	3.01 ^b^	3.63 ^a^	0.074	<0.01	<0.01	<0.01

Values with dissimilar lowercase letters in a given row represent significant differences (*p* < 0.05). Similar letters, or absence of letters, reflect no significant change (*p* > 0.05). Each group contains 16 replicates (*n* = 16).

**Table 3:** Effects of replacing soybean meal with yeast powder on the body condition of weaned pigs.

Items	Treatments				SEM	*p*-Value		
	CON	LYP	MYP	HYP		ANOVA	Linear	Quadratic
body height/cm	46.06 ^a^	46.00 ^a^	44.88 ^ab^	43.81 ^b^	0.252	0.002	<0.01	<0.01
stem length/cm	73.00 ^c^	85.19 ^a^	84.94 ^a^	80.63 ^b^	0.712	<0.01	<0.01	<0.01
bust/cm	75.81 ^a^	72.81 ^b^	70.69 ^c^	70.63 ^c^	0.414	<0.01	<0.01	<0.01

Values with dissimilar lowercase letters in a given row represent significant differences (*p* < 0.05). Similar letters, or absence of letters, reflect no significant change (*p* > 0.05). Each group contains 16 replicates (*n* = 16).

**Table 4:** Effect of replacing soybean meal with yeast powder on the apparent nutrient digestibility in weaned pigs.

Items	Treatments				SEM	*p*-Value		
	CON	LYP	MYP	HYP		ANOVA	Linear	Quadratic
CP/%	84.72	81.71	80.87	81.54	0.619	0.113	0.058	0.045
DM/%	87.78 ^a^	83.35 ^b^	82.33 ^b^	82.21 ^b^	0.640	<0.01	<0.01	<0.01
Ash/%	52.52 ^a^	38.21 ^bc^	32.85 ^c^	42.71 ^b^	2.078	<0.01	0.058	<0.01

Values with dissimilar lowercase letters in a given row represent significant differences (*p* < 0.05). Similar letters, or absence of letters, reflect no significant change (*p* > 0.05). Each group contains 4 replicates (*n* = 4).

**Table 5:** Effect of replacing soybean meal with yeast powder on serum calcium and phosphorus metabolism indexes in weaned pigs.

Items	Treatments				SEM	*p*-Value		
	CON	LYP	MYP	HYP		ANOVA	Linear	Quadratic
Ca, mmol/L	0.12	0.20	0.17	0.19	0.01	0.125	0.100	0.240
P, mmol/L	2.71	2.50	2.78	2.61	0.06	0.372	0.955	0.859
HYP, µg/mL	29.19 ^a^	29.14 ^a^	22.26 ^b^	20.65 ^b^	1.26	<0.01	<0.01	0.693
CT, pg/mL	15.08 ^c^	19.20 ^bc^	19.84 ^b^	25.25 ^a^	1.05	<0.01	<0.01	0.651
PTH, pg/mL	343.03 ^a^	289.78 ^b^	264.40 ^bc^	231.18 ^c^	11.04	<0.01	<0.01	0.442
ALP, U/mL	1.95 ^c^	2.48 ^bc^	3.70 ^ab^	4.73 ^a^	0.33	<0.01	<0.01	0.616

Values with dissimilar lowercase letters in a given row represent significant differences (*p* < 0.05). Similar letters, or absence of letters, reflect no significant change (*p* > 0.05). Each group contains 5 replicates (*n* = 5).

**Table 6:** Effect of replacing soybean meal with yeast powder on serum biochemical indexes in weaned pigs.

Items	Treatments				SEM	*p*-Value		
	CON	LYP	MYP	HYP		ANOVA	Linear	Quadratic
BUN, mmol/L	1.94 ^c^	2.51 ^b^	3.57 ^a^	3.19 ^ab^	0.18	<0.01	<0.01	0.054
GLU, mmol/L	0.15 ^a^	0.11 ^bc^	0.13 ^b^	0.10 ^c^	0.004	<0.01	<0.01	0.168
ALB, g/L	28.12 ^a^	28.43 ^a^	25.84 ^ab^	23.12 ^b^	0.64	<0.01	<0.01	0.111
TG, mmol/L	0.33 ^c^	0.49 ^bc^	0.61 ^ab^	0.87 ^a^	0.05	<0.01	<0.01	0.473
TP, g/L	73.36	72.81	68.92	72.43	0.85	0.254	0.378	0.234
T-CHO, mmol/L	2.97	3.35	2.86	3.68	0.15	0.175	0.202	0.432

Values with dissimilar lowercase letters in a given row represent significant differences (*p* < 0.05). Similar letters, or absence of letters, reflect no significant change (*p* > 0.05). Each group contains 5 replicates (*n* = 5).

**Table 7:** Effect of replacing soybean meal with yeast powder on serum antioxidant indexes in weaned pigs.

Items	Treatments				SEM	*p*-Value		
	CON	LYP	MYP	HYP		ANOVA	Linear	Quadratic
T-AOC, U/mL	1.76 ^b^	1.80 ^b^	1.87 ^a^	1.88 ^a^	0.01	<0.01	<0.01	0.482
T-SOD, U/mL	41.27	45.74	46.19	41.90	1.79	0.711	0.891	0.258
GSH-Px, U/mL	624.00 ^b^	634.40 ^b^	652.80 ^b^	858.40 ^a^	24.21	<0.01	<0.01	<0.01
CAT, U/mL	4.65	2.99	2.56	3.91	0.58	0.276	0.476	0.093
MDA, mmol/L	2.73	2.43	3.10	3.86	0.23	0.153	0.054	0.239

Values with dissimilar lowercase letters in a given row represent significant differences (*p* < 0.05). Similar letters, or absence of letters, reflect no significant change (*p* > 0.05). Each group contains 5 replicates (*n* = 5).

## Data Availability

The raw data for 16S rDNA sequencing can be accessed at NCBI with the accession number PRJNA985574 (https://www.ncbi.nlm.nih.gov/bioproject/prjna985574, accessed on 15 April 2026). Additional raw data supporting the findings of this study are available from the first author and the corresponding author upon request. Raw data are available by contacting the corresponding author.
